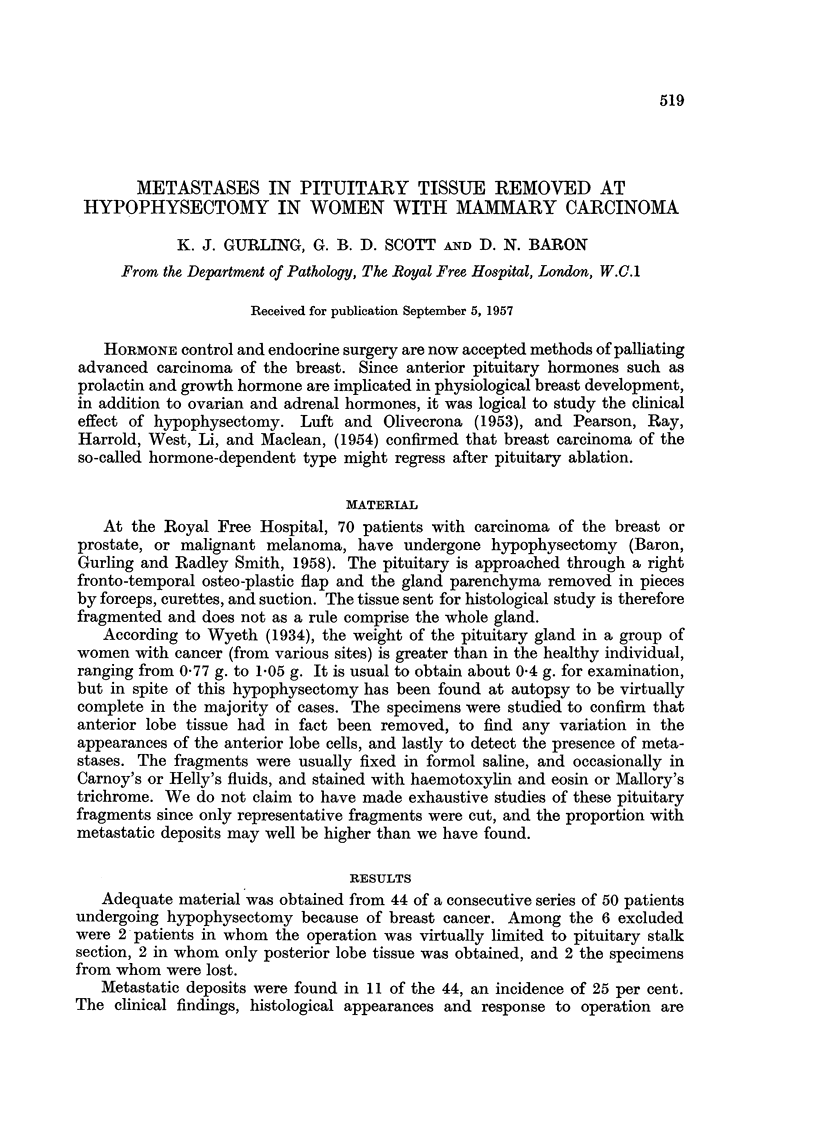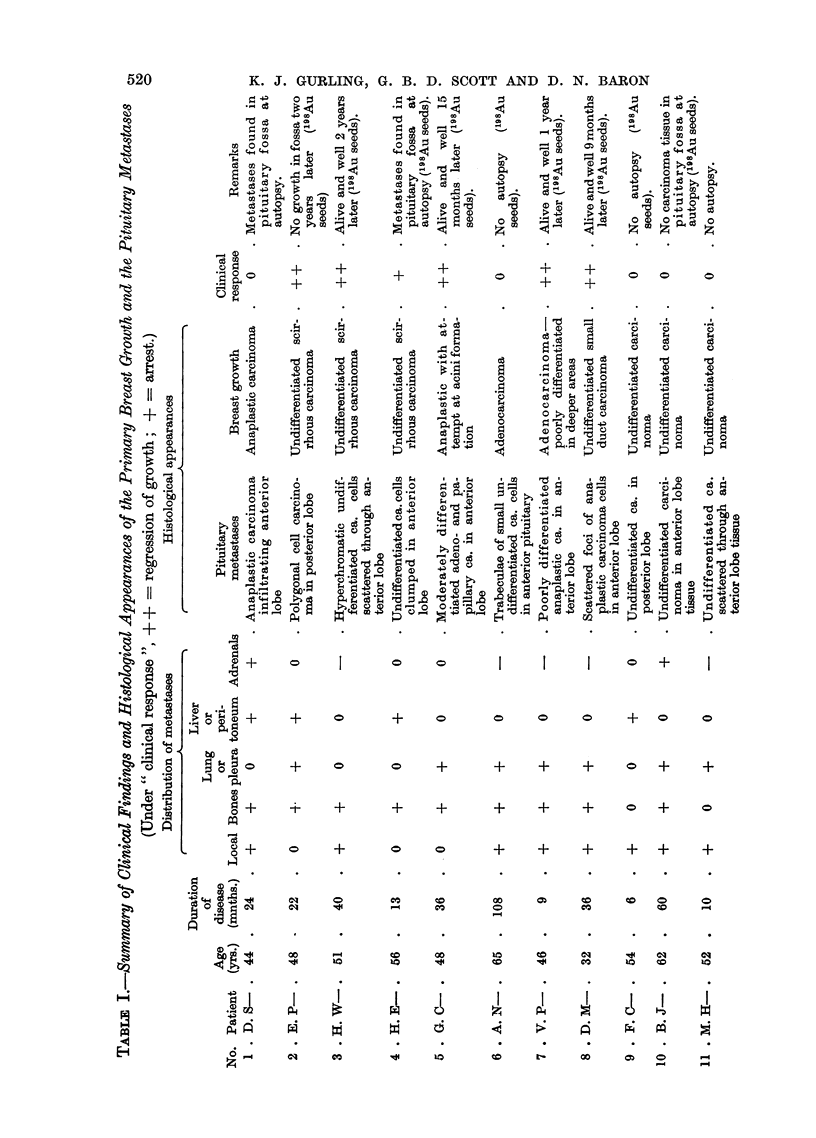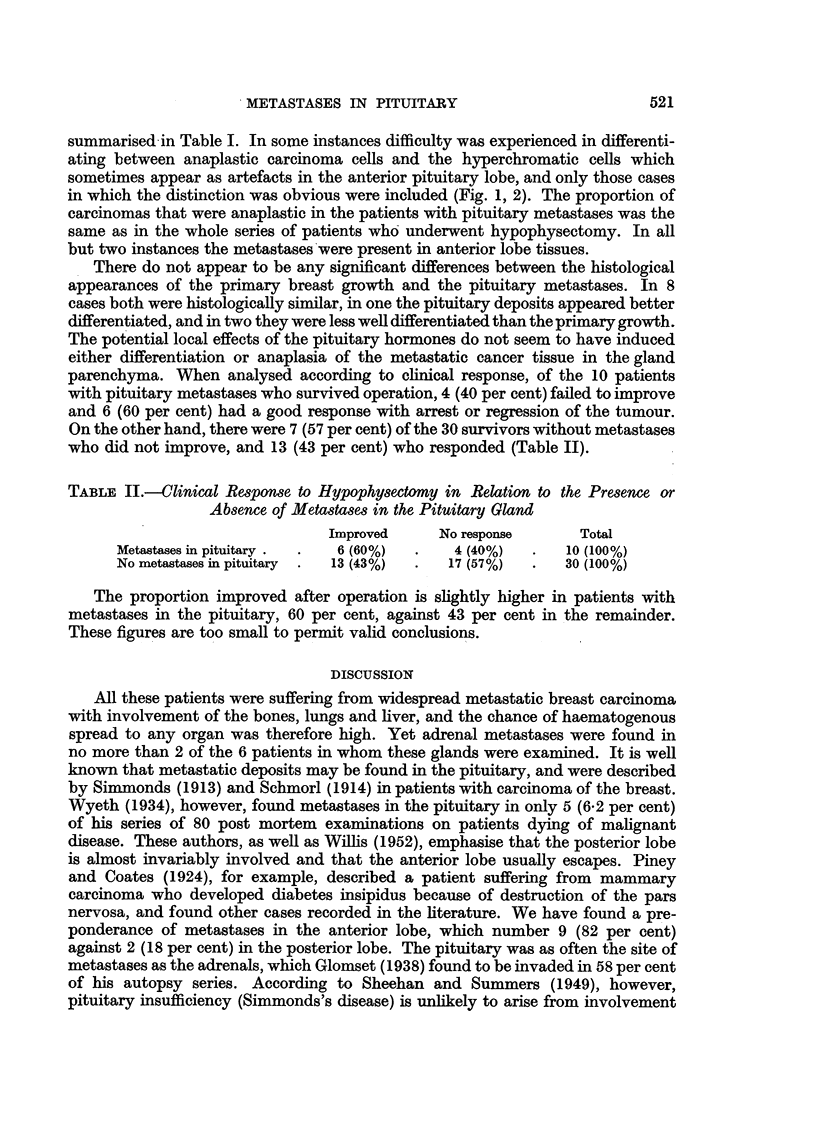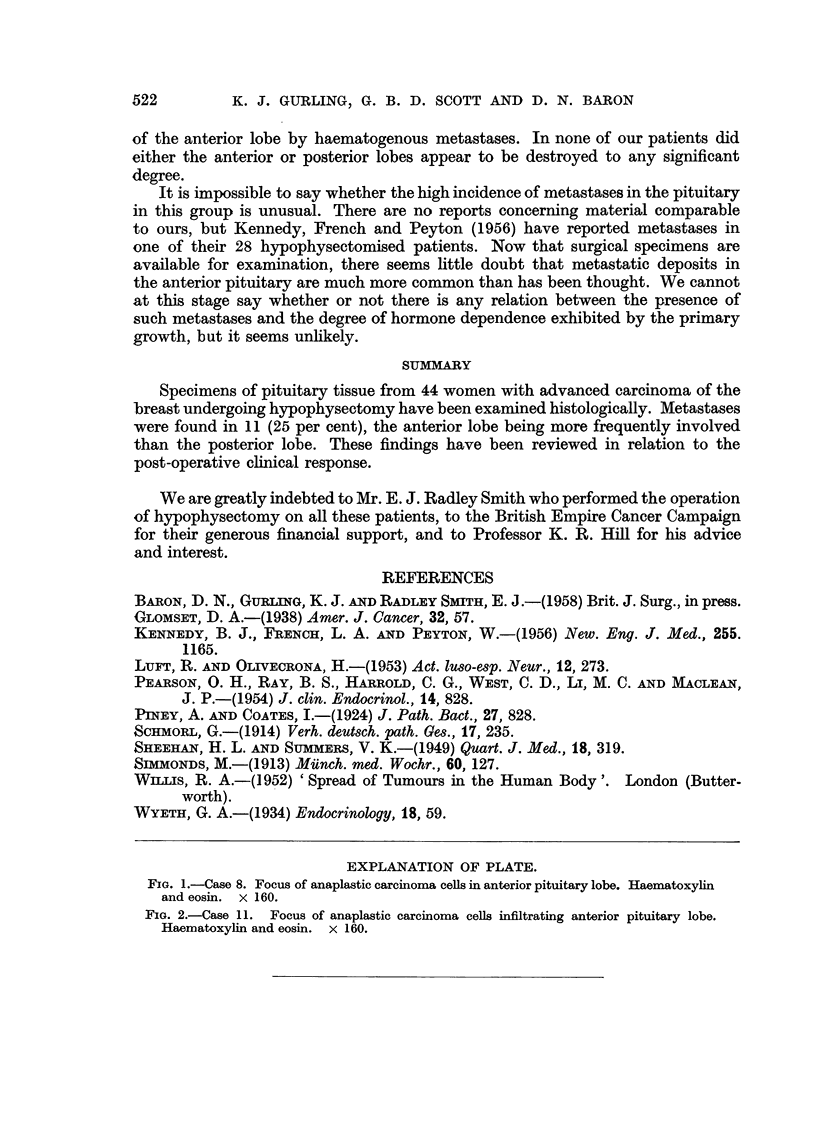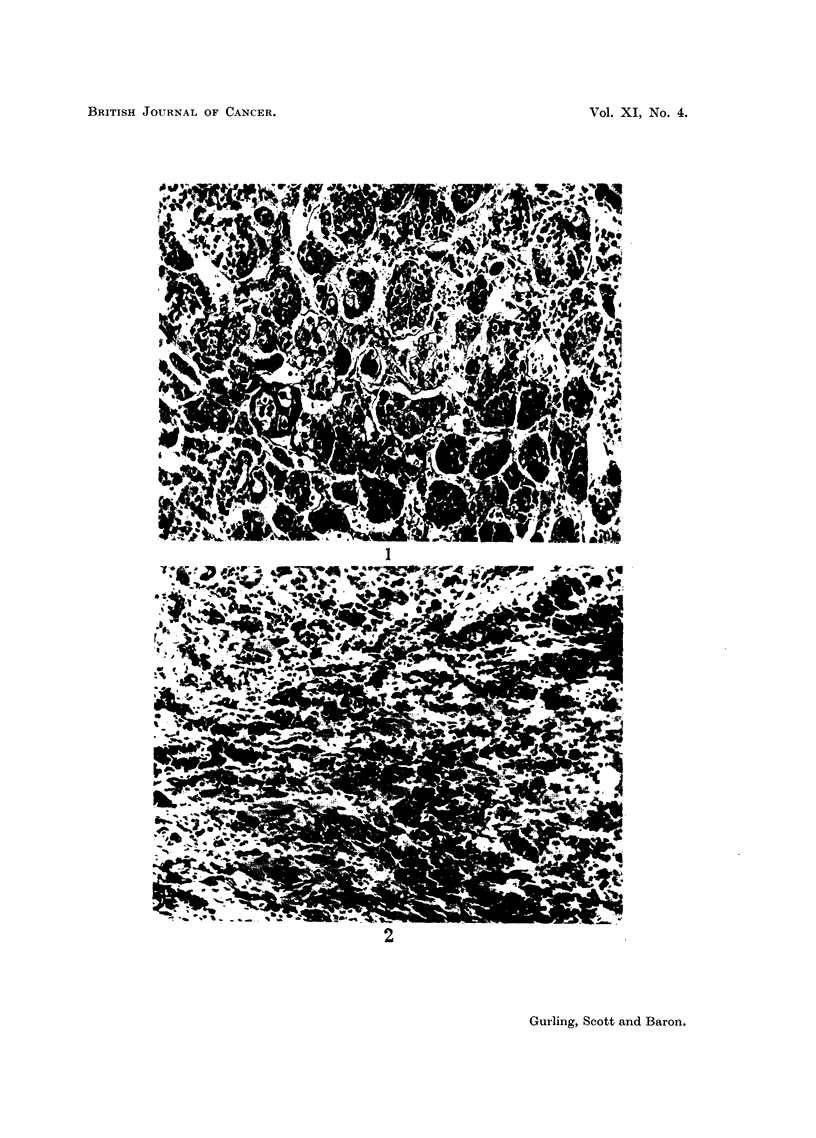# Metastases in Pituitary Tissue Removed at Hypophysectomy in Women with Mammary Carcinoma

**DOI:** 10.1038/bjc.1957.63

**Published:** 1957-12

**Authors:** K. J. Gurling, G. B. D. Scott, D. N. Baron

## Abstract

**Images:**


### METASTASES IN PITUITARY TISSUE REMOVED AT HYPOPHYSECTOMY IN WOMEN WITH MAMMARY CARCINOMA

K. J. GURLING, G. B. D. SCOTT AND D. N. BARON

*From the Department of Pathology, The Royal Free Hospital, London, W.C.1*

Received for publication September 5, 1957

HORMONE control and endocrine surgery are now accepted methods of palliating advanced carcinoma of the breast. Since anterior pituitary hormones such as prolactin and growth hormone are implicated in physiological breast development, in addition to ovarian and adrenal hormones, it was logical to study the clinical effect of hypophysectomy. Luft and Olivecrona (1953), and Pearson, Ray, Harrold, West, Li, and Maclean, (1954) confirmed that breast carcinoma of the so-called hormone-dependent type might regress after pituitary ablation.

### MATERIAL

At the Royal Free Hospital, 70 patients with carcinoma of the breast or prostate, or malignant melanoma, have undergone hypophysectomy (Baron, Gurling and Radley Smith, 1958). The pituitary is approached through a right fronto-temporal osteo-plastic flap and the gland parenchyma removed in pieces by forceps, curettes, and suction. The tissue sent for histological study is therefore fragmented and does not as a rule comprise the whole gland.

According to Wyeth (1934), the weight of the pituitary gland in a group of women with cancer (from various sites) is greater than in the healthy individual, ranging from 0·77 g. to 1·05 g. It is usual to obtain about 0·4 g. for examination, but in spite of this hypophysectomy has been found at autopsy to be virtually complete in the majority of cases. The specimens were studied to confirm that anterior lobe tissue had in fact been removed, to find any variation in the appearances of the anterior lobe cells, and lastly to detect the presence of metastases. The fragments were usually fixed in formol saline, and occasionally in Carnoy's or Helly's fluids, and stained with haemotoxylin and eosin or Mallory's trichrome. We do not claim to have made exhaustive studies of these pituitary fragments since only representative fragments were cut, and the proportion with metastatic deposits may well be higher than we have found.

### RESULTS

Adequate material was obtained from 44 of a consecutive series of 50 patients undergoing hypophysectomy because of breast cancer. Among the 6 excluded were 2 patients in whom the operation was virtually limited to pituitary stalk section, 2 in whom only posterior lobe tissue was obtained, and 2 the specimens from whom were lost.

Metastatic deposits were found in 11 of the 44, an incidence of 25 per cent. The clinical findings, histological appearances and response to operation are

TABLE I.—*Summary of Clinical Findings and Histological Appearances of the Primary Breast Growth and the Pituitary Metastases*

(Under "clinical response", ++ = regression of growth ; + = arrest.)

| | | | | Distribution of metastases | | | | | Histological appearances | | | |
|---|---|---|---|---|---|---|---|---|---|---|---|---|
| No. | Patient | Age (yrs.) | Duration of disease (mnths.) | Local | Bones | Lung or pleura | Liver or peri-toneum | Adrenals | Pituitary metastases | Breast growth | Clinical response | Remarks |
| 1 | D. S— | 44 | 24 | + | + | 0 | + | + | Anaplastic carcinoma infiltrating anterior lobe | Anaplastic carcinoma | 0 | Metastases found in pituitary fossa at autopsy. |
| 2 | E. P— | 48 | 22 | 0 | + | + | + | 0 | Polygonal cell carcinoma in posterior lobe | Undifferentiated scirrhous carcinoma | ++ | No growth in fossa two years later ($^{198}$Au seeds) |
| 3 | H. W— | 51 | 40 | + | + | 0 | 0 | — | Hyperchromatic undifferentiated ca. cells scattered through anterior lobe | Undifferentiated scirrhous carcinoma | ++ | Alive and well 2 years later ($^{198}$Au seeds). |
| 4 | H. E— | 56 | 13 | 0 | + | 0 | + | 0 | Undifferentiated ca. cells clumped in anterior lobe | Undifferentiated scirrhous carcinoma | + | Metastases found in pituitary fossa at autopsy ($^{198}$Au seeds). |
| 5 | G. C— | 48 | 36 | 0 | + | + | 0 | 0 | Moderately differentiated adeno- and papillary ca. in anterior lobe | Anaplastic with attempt at acini formation | ++ | Alive and well 15 months later ($^{198}$Au seeds). |
| 6 | A. N— | 65 | 108 | + | + | + | 0 | — | Trabeculae of small undifferentiated ca. cells in anterior pituitary | Adenocarcinoma | 0 | No autopsy ($^{198}$Au seeds). |
| 7 | V. P— | 46 | 9 | + | + | + | 0 | — | Poorly differentiated anaplastic ca. in anterior lobe | Adenocarcinoma—poorly differentiated in deeper areas | ++ | Alive and well 1 year later ($^{198}$Au seeds). |
| 8 | D. M— | 32 | 36 | + | + | + | 0 | — | Scattered foci of anaplastic carcinoma cells in anterior lobe | Undifferentiated small duct carcinoma | ++ | Alive and well 9 months later ($^{198}$Au seeds). |
| 9 | F. C— | 54 | 6 | + | 0 | 0 | + | 0 | Undifferentiated ca. in posterior lobe | Undifferentiated carcinoma | 0 | No autopsy ($^{198}$Au seeds). |
| 10 | B. J— | 62 | 60 | + | + | + | 0 | + | Undifferentiated carcinoma in anterior lobe tissue | Undifferentiated carcinoma | 0 | No carcinoma tissue in pituitary fossa at autopsy ($^{198}$Au seeds). |
| 11 | M. H— | 52 | 10 | + | 0 | + | 0 | — | Undifferentiated ca. scattered through anterior lobe tissue | Undifferentiated carcinoma | 0 | No autopsy. |

summarised in Table I. In some instances difficulty was experienced in differentiating between anaplastic carcinoma cells and the hyperchromatic cells which sometimes appear as artefacts in the anterior pituitary lobe, and only those cases in which the distinction was obvious were included (Fig. 1, 2). The proportion of carcinomas that were anaplastic in the patients with pituitary metastases was the same as in the whole series of patients who underwent hypophysectomy. In all but two instances the metastases were present in anterior lobe tissues.

There do not appear to be any significant differences between the histological appearances of the primary breast growth and the pituitary metastases. In 8 cases both were histologically similar, in one the pituitary deposits appeared better differentiated, and in two they were less well differentiated than the primary growth. The potential local effects of the pituitary hormones do not seem to have induced either differentiation or anaplasia of the metastatic cancer tissue in the gland parenchyma. When analysed according to clinical response, of the 10 patients with pituitary metastases who survived operation, 4 (40 per cent) failed to improve and 6 (60 per cent) had a good response with arrest or regression of the tumour. On the other hand, there were 7 (57 per cent) of the 30 survivors without metastases who did not improve, and 13 (43 per cent) who responded (Table II).

TABLE II.—*Clinical Response to Hypophysectomy in Relation to the Presence or Absence of Metastases in the Pituitary Gland*

| | Improved | No response | Total |
|---|---|---|---|
| Metastases in pituitary | 6 (60%) | 4 (40%) | 10 (100%) |
| No metastases in pituitary | 13 (43%) | 17 (57%) | 30 (100%) |

The proportion improved after operation is slightly higher in patients with metastases in the pituitary, 60 per cent, against 43 per cent in the remainder. These figures are too small to permit valid conclusions.

### DISCUSSION

All these patients were suffering from widespread metastatic breast carcinoma with involvement of the bones, lungs and liver, and the chance of haematogenous spread to any organ was therefore high. Yet adrenal metastases were found in no more than 2 of the 6 patients in whom these glands were examined. It is well known that metastatic deposits may be found in the pituitary, and were described by Simmonds (1913) and Schmorl (1914) in patients with carcinoma of the breast. Wyeth (1934), however, found metastases in the pituitary in only 5 (6·2 per cent) of his series of 80 post mortem examinations on patients dying of malignant disease. These authors, as well as Willis (1952), emphasise that the posterior lobe is almost invariably involved and that the anterior lobe usually escapes. Piney and Coates (1924), for example, described a patient suffering from mammary carcinoma who developed diabetes insipidus because of destruction of the pars nervosa, and found other cases recorded in the literature. We have found a preponderance of metastases in the anterior lobe, which number 9 (82 per cent) against 2 (18 per cent) in the posterior lobe. The pituitary was as often the site of metastases as the adrenals, which Glomset (1938) found to be invaded in 58 per cent of his autopsy series. According to Sheehan and Summers (1949), however, pituitary insufficiency (Simmonds's disease) is unlikely to arise from involvement of the anterior lobe by haematogenous metastases. In none of our patients did either the anterior or posterior lobes appear to be destroyed to any significant degree.

It is impossible to say whether the high incidence of metastases in the pituitary in this group is unusual. There are no reports concerning material comparable to ours, but Kennedy, French and Peyton (1956) have reported metastases in one of their 28 hypophysectomised patients. Now that surgical specimens are available for examination, there seems little doubt that metastatic deposits in the anterior pituitary are much more common than has been thought. We cannot at this stage say whether or not there is any relation between the presence of such metastases and the degree of hormone dependence exhibited by the primary growth, but it seems unlikely.

SUMMARY

Specimens of pituitary tissue from 44 women with advanced carcinoma of the breast undergoing hypophysectomy have been examined histologically. Metastases were found in 11 (25 per cent), the anterior lobe being more frequently involved than the posterior lobe. These findings have been reviewed in relation to the post-operative clinical response.

We are greatly indebted to Mr. E. J. Radley Smith who performed the operation of hypophysectomy on all these patients, to the British Empire Cancer Campaign for their generous financial support, and to Professor K. R. Hill for his advice and interest.

### REFERENCES

BARON, D. N., GURLING, K. J. AND RADLEY SMITH, E. J.—(1958) Brit. J. Surg., in press.
GLOMSET, D. A.—(1938) *Amer. J. Cancer*, **32**, 57.
KENNEDY, B. J., FRENCH, L. A. AND PEYTON, W.—(1956) *New. Eng. J. Med.*, **255**. 1165.
LUFT, R. AND OLIVECRONA, H.—(1953) *Act. luso-esp. Neur.*, **12**, 273.
PEARSON, O. H., RAY, B. S., HARROLD, C. G., WEST, C. D., LI, M. C. AND MACLEAN, J. P.—(1954) *J. clin. Endocrinol.*, **14**, 828.
PINEY, A. AND COATES, I.—(1924) *J. Path. Bact.*, **27**, 828.
SCHMORL, G.—(1914) *Verh. deutsch. path. Ges.*, **17**, 235.
SHEEHAN, H. L. AND SUMMERS, V. K.—(1949) *Quart. J. Med.*, **18**, 319.
SIMMONDS, M.—(1913) *Münch. med. Wochr.*, **60**, 127.
WILLIS, R. A.—(1952) ' Spread of Tumours in the Human Body '. London (Butterworth).
WYETH, G. A.—(1934) *Endocrinology*, **18**, 59.

---

EXPLANATION OF PLATE.

FIG. 1.—Case 8. Focus of anaplastic carcinoma cells in anterior pituitary lobe. Haematoxylin and eosin. × 160.

FIG. 2.—Case 11. Focus of anaplastic carcinoma cells infiltrating anterior pituitary lobe. Haematoxylin and eosin. × 160.

1

2

Gurling, Scott and Baron.